# New York Register of Medicine and Pharmacy

**Published:** 1850-10

**Authors:** 


*The New York Register of Medicine and Pharmacy, Edited by C. D. Griswold, M. D.*—The above is the title of a semi-monthly journal, published in the city of New York. We have received Nos. 2 and 3, and judging from these, it is conducted with much ability. Each No. contains several valuable and interesting papers, besides a number of spiritedly written editorials. We wish the Register abundant success.

